# mTORopathies in Epilepsy and Neurodevelopmental Disorders: The Future of Therapeutics and the Role of Gene Editing

**DOI:** 10.3390/cells14090662

**Published:** 2025-04-30

**Authors:** Marina Ottmann Boff, Fernando Antônio Costa Xavier, Fernando Mendonça Diz, Júlia Budelon Gonçalves, Laura Meireles Ferreira, Jean Zambeli, Douglas Bottega Pazzin, Thales Thor Ramos Previato, Helena Scartassini Erwig, João Ismael Budelon Gonçalves, Fernanda Thays Konat Bruzzo, Daniel Marinowic, Jaderson Costa da Costa, Gabriele Zanirati

**Affiliations:** 1 Brain Institute of Rio Grande do Sul (BraIns), Pontifical Catholic University of Rio Grande do Sul (PUCRS), Porto Alegre 90610-000, RS, Brazil; ottmann.marina@edu.pucrs.br (M.O.B.); fxavier@pucrs.br (F.A.C.X.); fernandomendoncadiz@gmail.com (F.M.D.); julybudelon@gmail.com (J.B.G.); laurameifer@gmail.com (L.M.F.); jeanzambeli@gmail.com (J.Z.); douglas.pazzin@edu.pucrs.br (D.B.P.); thales.previato@edu.pucrs.br (T.T.R.P.); helena.scartassini@edu.pucrs.br (H.S.E.); joaoismaelbudelon@gmail.com (J.I.B.G.); fbruzzo94@gmail.com (F.T.K.B.); daniel.marinowic@pucrs.br (D.M.); jcc@pucrs.br (J.C.d.C.); 2 School of Medicine, Pontifical Catholic University of Rio Grande do Sul (PUCRS), Porto Alegre 90619-900, RS, Brazil; 3 Graduate Program in Medicine and Health Sciences, School of Medicine, Pontifical Catholic University of Rio Grande do Sul (PUCRS), Porto Alegre 90619-900, RS, Brazil; 4 School of Medicine, University of the Valley of the Rio dos Sinos (UNISINOS), São Leopoldo 93022-750, RS, Brazil; 5 Graduate Program in Pediatrics and Child Health, School of Medicine, Pontifical Catholic University of Rio Grande do Sul (PUCRS), Porto Alegre 90619-900, RS, Brazil; 6 Graduate Program in Biomedical Gerontology, School of Medicine, Pontifical Catholic University of Rio Grande do Sul (PUCRS), Porto Alegre 90619-900, RS, Brazil; 7 School of Health and Life, Pontifical Catholic University of Rio Grande do Sul (PUCRS), Porto Alegre 90619-900, RS, Brazil

**Keywords:** gene therapy, mTOR, mTORopathy, epilepsy, extracellular vesicles, exosomes, CRISPR/Cas9

## Abstract

mTORopathies represent a group of neurodevelopmental disorders linked to dysregulated mTOR signaling, resulting in conditions such as tuberous sclerosis complex, focal cortical dysplasia, hemimegalencephaly, and Smith–Kingsmore Syndrome. These disorders often manifest with epilepsy, cognitive impairments, and, in some cases, structural brain anomalies. The mTOR pathway, a central regulator of cell growth and metabolism, plays a crucial role in brain development, where its hyperactivation leads to abnormal neuroplasticity, tumor formation, and heightened neuronal excitability. Current treatments primarily rely on mTOR inhibitors, such as rapamycin, which reduce seizure frequency and tumor size but fail to address underlying genetic causes. Advances in gene editing, particularly via CRISPR/Cas9, offer promising avenues for precision therapies targeting the genetic mutations driving mTORopathies. New delivery systems, including viral and non-viral vectors, aim to enhance the specificity and efficacy of these therapies, potentially transforming the management of these disorders. While gene editing holds curative potential, challenges remain concerning delivery, long-term safety, and ethical considerations. Continued research into mTOR mechanisms and innovative gene therapies may pave the way for transformative, personalized treatments for patients affected by these complex neurodevelopmental conditions.

## 1. Introduction to the mTOR Complex and Its Relevance in Neurodevelopment

**Figure 1:**
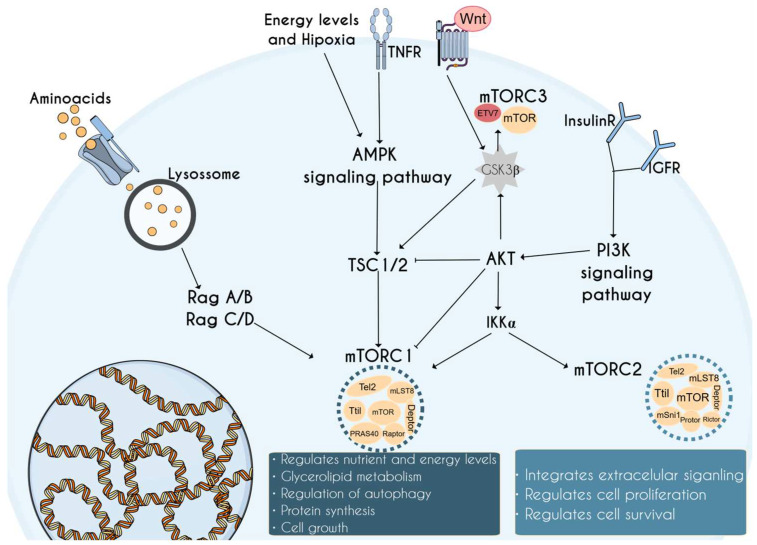
The mTOR pathway. mTORC1 regulates nutrient and energy levels, lipid metabolism, autophagy, and cell growth, while mTORC2 integrates extracellular signals, regulating cell proliferation and survival. Little is known about mTORC3; however, ETV7 expression stimulates its transcription. The figure demonstrates the complexity of mTOR signaling and its central role in coordinating cellular responses to both internal and external stimuli.

The PI3K/AKT/mTOR signaling pathway (Figure 1) is a fundamental intracellular network that integrates multiple other signaling pathways. Together, these pathways are crucial for maintaining homeostasis by regulating key metabolic and cellular processes such as growth, proliferation, and autophagy across various tissues. Additionally, they are involved in broader physiological functions, including the organism’s adaptive response to stress [1]. Among the key components of this network, the mechanistic target of rapamycin (mTOR) [2] pathway stands out for its central role in controlling cellular metabolism. It regulates essential processes like catabolism, immune responses, autophagy, survival, proliferation, and migration—all of which are vital to preserving cellular and systemic equilibrium [3].

The mechanism of action of the mTOR pathway is intricately tied to its target protein, rapamycin, a compound discovered during research on a strain of *Streptomyces* isolated from soil samples collected on Easter Island (Rapa Nui), specifically from the mycelium of *Streptomyces hygroscopicus* [4]. The discovery of rapamycin and its corresponding target of rapamycin (TOR) was pivotal in advancing the molecular and physiological understanding of what is now known as the mTOR pathway. This pathway is defined by three distinct complexes: mTORC1, mTORC2, and mTORC3 [5]. Despite their differing functions, mTORC1 and mTORC2 share the common kinase mTOR and its constitutive binding partner mLST8, resulting in similar regulatory mechanisms [6]. mTORC1 plays a crucial role in coordinating cellular growth and tissue development by regulating nutrient and energy levels within the cell and orchestrating both catabolic and anabolic processes [7]. The activity of mTORC1 is highly responsive to nutrient availability, particularly amino acids, while glucose, lipids, hormones, and growth factors also serve as critical regulators of its function [8,9].

Rapamycin has been shown to act as a specific inhibitor of the mTORC1 complex, a characteristic not observed in the mTORC2 and mTORC3 complexes, which do not exhibit an affinity for rapamycin [10]. mTORC2 plays a distinct role by integrating extracellular signals, such as growth factors and cytokines, to promote the activation of mTORC1, cellular proliferation, and survival. This is achieved through the direct phosphorylation of Akt, a protein kinase that, once activated, regulates cellular metabolism, survival, and stress responses [11]. Research into the regulatory mechanisms of the mTOR pathway has been critical for understanding how disruptions in this pathway contribute to various physiological processes, and how they are associated with a wide range of diseases, including cancer, diabetes, Parkinson’s disease, and Alzheimer’s disease [12,13,14].

mTOR inhibitors also play a crucial role in treating the neuropsychiatric symptoms of mTORopathies, such as tuberous sclerosis, by modulating the mTOR pathway, which is dysregulated in these conditions. Studies involving children aged 12 to 24 months, using the Bayley Scales of Infant and Toddler Development III (BSID-III), have shown significant improvements in the severity of neuropsychiatric disorders [15]. These inhibitors are particularly relevant because the mTOR pathway is involved in processes such as synaptic plasticity, autophagy, and cell growth regulation, whose dysregulation is associated with neurological and psychiatric disorders. Evidence suggests that modulating this pathway can be beneficial in conditions such as epilepsy, autism, mood disorders, schizophrenia, and even drug addiction, as in the case of methamphetamine. By restoring normal mTOR pathway function, these inhibitors can alleviate neuropsychiatric symptoms, improve cognitive function, and reduce neurodegenerative processes, thus emerging as a promising therapeutic approach [16]. Studies conducted in murine models demonstrate that the use of mTORC1 inhibitors, such as sirolimus and everolimus, significantly improves clinical symptoms associated with autism, while also enhancing cognitive function and reducing neuropsychological deficits [17]. Furthermore, antipsychotic drugs, including olanzapine, aripiprazole, and sertindole, have been observed to exert mTOR inhibitory effects, which might contribute to their therapeutic efficacy by maintaining mTOR pathway stability [18,19]. In addition, mTOR inhibitors modulate synaptic plasticity and cognitive function in a dose-dependent manner [20], whereby moderate inhibition may enhance learning and memory [21], while excessive suppression can disrupt synaptic homeostasis [22], underscoring the need for further clinical research.

First proposed by Luo et al. (2015) [23] and later characterized by Harwood et al. (2018) [24], mTORC3 is a novel mTOR complex that emerges in response to ETV7 expression, which plays a crucial role in its assembly and activation. This complex exhibit heightened kinase activity, promoting cellular proliferation and contributing to rapamycin resistance, although the precise molecular mechanisms driving this resistance remain to be fully elucidated [5,25]. Despite these initial insights, information regarding other functional and structural characteristics of mTORC3 remains limited. Further investigation into this complex could be crucial for advancing our understanding of mTORopathies, particularly in uncovering new therapeutic targets or mechanisms underlying drug resistance in these disorders.

In the nervous system, mTOR plays pivotal roles at crucial stages of neural development and regulation. Molecules such as brain-derived neurotrophic factor (BDNF), insulin, insulin-like growth factor 1 (IGF-1), vascular endothelial growth factor (VEGF), ciliary neurotrophic factor (CNTF), and glutamate serve as key modulators of the mTOR pathway in this context. These molecules influence neuronal electrophysiological activity and regulate the synthesis of dendritic proteins [26,27]. The activation of the mTOR pathway by brain-derived neurotrophic factor (BDNF) occurs due to the phosphorylation of the proteins 4EBP, p70S6K, and S6, which promotes the production of local proteins and the activation of neuronal dendrites [26]. Another similar process occurs in the presence of IGF-1. When insulin-like growth factor 1 (IGF-1) binds to its receptors (IGF1R), a series of cascades occur that result in the activation of the mTORC2 complex, which regulates the cell cycle [28].

Excessive activation of the mTOR pathway is associated with a cascade of detrimental effects, including structural alterations and neuronal damage, inhibition of autophagy, increased neuroinflammation, and heightened neuronal excitability—all of which are strongly linked to epilepsy [29]. Elevated mTOR signaling, due to the loss of TSC1/2 or PTEN, induces significant changes in neuronal architecture and differentiation, a hallmark associated with the development of primary central nervous system lymphoma [30,31]. Neurons lacking TSC1/2 exhibit defects in cellular maturation, characterized by the abnormal formation of multiple axons. Patients with loss-of-function mutations in the PTEN gene, a negative regulator of mTOR signaling, are predisposed to developing macrocephaly, autism spectrum disorder (ASD), seizures, and intellectual disability [32]. PTEN regulates the PI3K/AKT/mTOR pathway by dephosphorylating PIP3 to PIP2, thereby reducing levels of activated AKT. The pathway is further modulated by mTORC2, which phosphorylates AKT. PTEN is essential for maintaining cellular homeostasis [33].

These findings have sparked extensive research into the connection between the mTOR pathway and neurological diseases, with the goal of uncovering the mechanisms driving these disorders and identifying novel therapeutic strategies to target and treat these conditions. The dysregulation of the mTOR pathway can have profound consequences, leading to abnormal neurodevelopment and metabolic disturbances, which are linked to a group of neurological disorders known as mTORopathies [34].

## 2. mTORopathies: mTOR-Related Disorders

mTORopathies are disorders influenced by the mTOR pathway, particularly through its hyperactivation. Many of these conditions are related to neurodevelopmental disorders such as tuberous sclerosis complex (TSC), focal cortical dysplasia type 2 (FCD2), hemimegalencephaly (HME), Smith–Kingsmore Syndrome (SKS), POLR2A-related syndrome associated with epilepsy and mental retardation, polyhydramnios, megalencephaly, symptomatic epilepsy (PMSE), PTEN syndrome, DEPDC5-related syndrome, and PI3K-related overgrowth syndrome (PROS). Dysregulation of mTOR signaling can lead to aberrant neuroplasticity, resulting in pathological changes in the structure and function of neural networks, which contribute to the formation of epileptogenic circuits. Hyperactivation of the mTOR pathway may cause neuronal hyperexcitability, increasing the likelihood of seizures in affected individuals. Additionally, mTORopathies are associated with tumor formation and malformations, which can give rise to cortical lesions, leaving focal areas prone to seizures [35].

Tuberous sclerosis complex (TSC) is a genetic disorder that arises from dysfunction in the mTOR pathway, and it is marked by the development of benign tumors in multiple organs, particularly in the brain. Histological examination of cortical tubers in individuals with TSC mutations reveals the presence of dysmorphic and giant cells [36]. The predominant pathophysiological mechanism involves mutations in the TSC1 or TSC2 genes, which disrupt the TSC complex’s ability to inhibit mTOR signaling. This loss of inhibition leads to a range of neurological manifestations, including seizures, refractory epilepsy, developmental delays, and behavioral issues [35]. Importantly, mTOR inhibitors have demonstrated efficacy in treating such tumors and reducing their size, as well as in alleviating neurological symptoms in TSC patients [37], thereby improving clinical outcomes and enhancing the quality of life for those undergoing such treatments.

Smith–Kingsmore Syndrome (SKS) is a rare genetic disorder resulting from either de novo or inherited gain-of-function mutations in the mTOR pathway [38], which interfere with the packing of the alpha-helix inhibitory domains and the FAT and kinase domains [39]. SKS is associated with megalencephaly and exhibits neuroanatomical abnormalities such as macrocephaly, epilepsy, intellectual disability, neurocognitive decline, and sleep disturbances. Extra-cerebral manifestations, including hypoglycemia, may also be present [40]. The variability in patient outcomes is influenced by the origin of mutations (somatic versus germinal), the extent of mosaicism, and the presence of extra-cerebral features [41]. These genetic variations can lead to diverse responses to pharmacological treatments, with some cases exhibiting refractory characteristics [38].

Focal cortical dysplasia type II (FCDII) is linked to somatic mutations in the mTOR pathway [42] and is characterized by disrupted cortical lamination and the presence of dysmorphic neurons [36]. Notably, subtype IIB displays balloon cells, which bear a resemblance to the cortical tubers found in TSC [36]. Patients with FCDII often experience refractory epilepsy and may present with autistic features [42]. In many cases, surgical resection of the affected cortical areas at an early age is necessary to effectively manage seizure activity [43].

Hemimegalencephaly (HME) is a malformation that histopathologically resembles FCD [44] and is defined by the presence of anomalous neuronal and glial cells within one hemisphere of the brain [45]. Histopathological features commonly include cytomegalic and dysmorphic neurons, along with balloon cells [46]. MRI findings typically reveal abnormalities such as pachygyria, polymicrogyria, ventricular dilation, and periventricular hyperintensities [46]. Clinically, HME is associated with epilepsy, intellectual disability, autistic features, and systemic disorders, including various dysplasias [47]. Additionally, HME can occur in conjunction with TSC, often resulting in a more severe clinical course and earlier onset of epilepsy [48].

PI3K-related overgrowth syndrome (PROS) is a group of diseases characterized by a gain of function in the PI3K gene, leading to hyperactivation of the PI3K/Akt/mTOR pathway [49]. PROS affects multiple systems, including vascular, musculoskeletal, and neurological systems. Neurological manifestations include megalencephaly syndromes, such as Megalencephaly–Capillary Malformation (MCAP or M-CM) and Dysplastic Megalencephaly (DMEG) [49,50]. The overlapping clinical features pose a diagnostic challenge, and genetic testing is further complicated by the involvement of multiple tissues [50].

PMSE syndrome, also known as Pretzel Syndrome, is caused by a homozygous truncating germline mutation in the *STE20-related kinase adaptor alpha* (*STRADA*) gene, an upstream regulator of mTORC1 [51,52]. An increased pool of radial glia associated with dysregulation of the mTOR pathway may contribute to the megalencephaly observed with this syndrome [51]. The CNS findings include macrocephaly, cognitive disability, epilepsy, subependymal dysplasias, and ventriculomegaly [53,54].

POLR2A-related syndrome associated with epilepsy and mental retardation is caused by specific variants of a mutation in the largest subunit of RNA polymerase II [55]. Common neurological phenotypes in affected patients include hypotonia, cerebellar and behavioral abnormalities, severe epilepsy, sleep disorders, and neurodevelopmental delay [55,56]. POLR2A and mTOR can be dysregulated simultaneously [57]; however, further investigations are needed to elucidate their mutual impact on their respective pathways.

Phosphatase and tensin gene (PTEN) plays a crucial role in various neuronal processes, including synaptic plasticity, NMDA/AMPA receptor responses, spine morphology, nuclear mechanisms, axon guidance, and neurite outgrowth regulation [58]. Loss-of-function mutations in PTEN lead to hyperactivation of both mTORC1 and mTORC2, which can contribute to epileptic activity [59,60]. Additionally, PTEN dysfunction is associated with clinical conditions such as autism spectrum disorder, Alzheimer’s and Parkinson’s diseases, and macrocephaly, highlighting its critical and complementary role within the PI3K/Akt/mTOR signaling pathway in neurons [58,60].

DEPDC5-related syndrome is an autosomal dominant disorder associated with epilepsy syndromes, developmental delays, intellectual disability, and autism spectrum disorder [61]. Its pathogenesis involves mTORC1 hyperactivation, as *DEPDC5* is a component of the GATOR1 complex, which functions as a negative regulator of mTORC1 [62]. Dysplastic and ectopic neurons may also be present in this syndrome [63]; however, brain MRIs appear normal in approximately 80% of cases [62], making clinical diagnosis challenging and necessitating molecular testing and precision medicine approaches.

## 3. Gene Editing in the Treatment of mTORopathies

Gene therapies represent medical interventions that modify a patient’s genetic material to prevent or treat diseases. These therapies aim to directly alter the gene responsible for a given condition, and their development has accelerated significantly since the advent of CRISPR/Cas9 gene-editing technology nearly a decade ago [64]. Since then, numerous studies have been conducted to explore gene therapies for various genetic disorders, with several receiving regulatory approval [65,66].

Despite its recent rise in prominence, gene therapy is a concept that dates back decades. The first recognized genetic therapy for a molecular disease was proposed by Linus Pauling and Harvey Itano in 1949, marking the beginning of the gene therapy era [67]. Their gene therapy for sickle-cell disease, known as CASGEVY, involved the ex vivo conversion of erythroid stem-cell β-globin subunits into γ-globin [67] by inactivating the BCL11A gene [66]. Following the discovery of DNA’s structure and functions [68,69], research groups began experimenting with the mechanisms used by viruses and bacteria to transfer genetic material. In 1973, the idea of using viral vectors to alter cellular function was first explored, and soon after, these approaches were tested in human patients [70,71]. The first attempt to use gene therapy with recombinant DNA in humans occurred in 1990 [72], followed by the approval of a clinical protocol in 1992 to introduce a foreign gene into humans, where tumor-infiltrating lymphocytes combined with interleukin-2 were used to suppress tumor growth [73].

China became the first country to approve gene therapy for clinical use in 2003, with Gendicine™ and Oncorine™ distributed without phase III clinical trial data. By 2008, Cerepro^®^ became the first adenoviral vector to complete a phase III trial, and in 2012, the European Medicines Agency (EMA) recommended Glybera, marking the first gene therapy product approved by the agency [74]. Today, numerous Advanced Therapy Medicinal Products (ATMPs) have received clinical approval. The EMA has approved 15 gene therapy ATMPs [75], while the U.S. Food and Drug Administration (FDA) has approved 38 cellular and gene therapy products [76].

Gene editing, once considered a novel therapeutic avenue, has now become a well-established tool for modeling diseases both in vitro and in vivo. In particular, mTORopathies—disorders arising from mutations in the mTOR pathway—have been extensively studied through these techniques, with tuberous sclerosis complex (TSC) serving as a prominent model. Mutations in TSC1 and TSC2, leading to loss of function, have prompted numerous research groups to develop knockout models targeting these genes [77]. Furthermore, Polyhydramnios–Megalencephaly–Symptomatic Epilepsy Syndrome (PMSE) has been modeled by silencing STRADA [78] and focal cortical dysplasia type II (FCDII) through a Depdc5 knockout [79]. Some autism spectrum disorder models have also explored PTEN knockout to replicate the Purkinje cell phenotype [80]. However, despite these advancements, no mTORopathy model fully captures the complete phenotype of a specific disease.

The development of these models typically begins with the patient’s genotype. For example, sequencing patient samples to identify specific mutations, such as those in TSC1 or TSC2, enables the creation of cell lines or animal models carrying the same mutation. Another promising approach in gene therapy is the use of induced pluripotent stem cells (iPSCs) derived from patients with the target disease, which retain the patient’s entire genotype [81].

Knockout mouse models for TSC1 and TSC2 have been extensively studied due to their effectiveness in replicating the TSC phenotype in select brain regions. One study on the TSC1 knockout mouse model investigated gene therapy to restore TSC1 activity, highlighting the potential of this therapeutic approach [82] (Table 1). Additionally, several research groups have examined gene therapy to assess its ability to improve mutant phenotypes in animal models of mTORopathies. For instance, TSC1 knockout models have shown amelioration following gene therapy targeting Rictor expression [32] and hamartin replacement [82,83]. Similarly, TSC2 knockout models have demonstrated positive outcomes with tuberin replacement [84].

Viral vectors (VVs), which deliver the desired genetic material, have been central to gene therapy since its inception, particularly for somatic modifications. These vectors remain among the most commonly used tools in gene editing due to their ability to provide long-term expression. However, long-term expression can sometimes lead to unintended consequences, such as off-target cleavage. As a result, gene-editing tools designed for short-term expression are often preferred to mitigate the risk of such effects [85].

Lipid-based delivery platforms, such as cell-derived vesicles, offer a shorter-term expression alternative compared with VVs. These platforms also reduce direct degradation and immune recognition, allowing them to circulate throughout the body until they are internalized by target cells [86]. Despite these advancements, concerns persist regarding the integration of genetic material from vectors, off-target effects, and long-term risks, which continue to make gene therapy a technique that requires careful consideration for safety [71,76].

In conclusion, gene therapy within the context of mTORopathies has been explored through various gene-editing methods, both for the development of disease models and for the investigation of novel therapeutic strategies.

**Table 1:** Gene therapy studies: organisms, targets, and editing methods across time.

Organism/Cell Type	Target	Editing Method	Year	Reference
Mice	STRADA	shRNA	2013	[78]
Mice	Depdc5	TALEN	2016	[79]
Mice	PTEN	Cre-Lox recombination	2016	[80]
Mice	Depdc5	Cre-Lox recombination	2018	[63]
Rat	Depdc5	CRISPR	2018	[87]
Mice	Depdc5	Cre-Lox recombination	2020	[88]
Mice	STRADA	shRNA	2010	[54]
Mice	TSC1	Rous sarcoma virus-based retroviral vectors	2001	[89]
Mice	TSC1	Cre-Lox recombination	2007	[90]
Mice	TSC1	Cre-Lox recombination	2012	[91]
Mice	TSC1	Cre-Lox recombination	2013	[92]
Mice	Rictor	Cre-Lox recombination	2020	[32]
Mice	TSC2	Cre-Lox recombination	2013	[93]
Mice	TSC1	Cre-Lox recombination	2011	[94]
Mice	RHEB	Plasmid construct	2019	[95]
hiPSCs	TSC2	CRISPR	2018	[96]
hiPSCs	TSC2	ZFN	2016	[97]
hESCs	PTEN	CRISPR	2019	[98]
N2a	TSC2/Depdc5	CRISPR	2021	[99]
Mice	TSC2	Cre-Lox recombination	2021	[84]
Mice	TSC1	Cre-Lox recombination	2019	[83]
Mice	TSC1	Cre-Lox recombination	2016	[82]

## 4. Available Types of Gene Editing

CRISPR-Cas9 is a groundbreaking genome-editing tool derived from a bacterial adaptive immune system that has been repurposed for precise genetic modifications in various organisms [100]. The system consists of two key components: a guide RNA (gRNA), which directs the Cas9 nuclease to a specific DNA sequence, and the Cas9 protein, which introduces a double-strand break at the targeted site [101]. This break is then repaired by the cell’s endogenous DNA repair mechanisms, either through error-prone non-homologous end joining (NHEJ) or high-fidelity homology-directed repair (HDR) [102]. The simplicity, efficiency, and specificity of CRISPR-Cas9 have revolutionized its application in biological research, enabling gene knockouts, insertions, and corrections, with profound implications for genetics research, disease modeling, and the development of gene therapies.

In the context of mTORopathies, CRISPR-Cas9 can be employed to either repair or knock out specific genes involved in the hyperactivation of the mTOR pathway. Gain-of-function mutations result in proteins that are overactive or hyper-responsive, leading to continuous stimulation of the mTOR pathway, which causes abnormal cell growth and other disease manifestations [3]. CRISPR-Cas9 can address these mutations by selectively targeting and either knocking out or precisely editing the mutated genes responsible for mTOR overactivation. In cancers with mTOR gain-of-function mutations, such as glioblastoma or renal cell carcinoma [103], CRISPR-Cas9 can be used to selectively knock out the mutated allele, thereby reducing excessive mTOR signaling, which could help control tumor growth driven by mTOR overactivation.

PIK3CD encodes the p110δ subunit of phosphoinositide 3-kinase delta (PI3Kδ), a key regulator in the PI3K–Akt–mTOR signaling pathway [104]. While PI3Kδ is predominantly expressed in immune cells, gain-of-function mutations can cause hyperactivation of the mTOR pathway, contributing to disorders like activated PI3K-δ syndrome (APDS) and certain cancers [105]. CRISPR-Cas9 knockout of PIK3CD in U87-MG cells has been shown to reduce migration, invasion, and colony formation abilities compared with parental cells [106]. Additionally, PIK3CD knockout has been shown to diminish tumorigenesis in nude mice [106]. RHEB encodes a GTPase that directly activates mTORC1 [107]. Targeting RHEB for knockout in cases of tuberous sclerosis or other neurological mTORopathies could prevent mTORC1 overactivation and slow the abnormal growth associated with these disorders. Furthermore, AKT1 encodes a protein kinase that acts upstream of mTOR and is activated in response to growth factors [108]. These examples illustrate how CRISPR-Cas9 knockout strategies can be applied to target hyperactive genes driving mTORopathies, offering potential avenues to restore normal mTOR signaling and reduce disease pathology.

As an alternative to traditional CRISPR-Cas9 technologies, the Base Editing technique, introduced in 2016, represents a promising approach for precise gene editing. This system employs a modified version of the Cas9 protein, termed Cas9 nickase, which induces a single-strand break in the DNA. Cas9 nickase is fused to a deaminase enzyme, which performs direct modifications of individual nucleotides, enabling the conversion of one base to another with high specificity [109]. Prime Editing, first introduced in 2019, also relies on Cas9 nickase, which is guided by the guiding RNA (pegRNA) to the target site. This pegRNA contains the sequence to be incorporated, serving as a template for the desired genetic modification. Additionally, a reverse transcriptase enzyme is fused to the Cas9 complex, facilitating the incorporation of the new genetic sequence by replacing the original DNA strand [110]. Due to the generation of a single-strand break, these editing tools minimize the risk of off-target mutations or large insertions and deletions (indels), a limitation commonly observed with CRISPR/Cas9 [111]. Furthermore, Prime Editing enables precise base substitutions, insertions, or deletions without requiring an external donor template for homologous recombination, as necessitated by homology-directed repair (HDR) [112].

The precision and versatility of Base Editing and Prime Editing offer new therapeutic possibilities for treating mTORopathies. Mutations in genes such as TSC1, TSC2, and MTOR itself, which lead to hyperactivation of the mTOR pathway, are central to the pathogenesis of diseases like tuberous sclerosis complex (TSC) and certain forms of cancer. By leveraging the ability of Base Editing to convert single nucleotide mutations and Prime Editing to introduce precise genetic modifications, it is possible to directly target and correct disease-causing mutations within these key regulatory genes. For instance, correcting loss-of-function mutations in TSC1 or TSC2 through these techniques could restore the proper inhibition of mTOR signaling, reducing aberrant cell growth and proliferation. Likewise, Prime Editing’s capacity for performing precise edits without inducing double-strand breaks or relying on homology-directed repair offers a safer and more efficient alternative to traditional gene-editing approaches. This targeted therapeutic potential could be transformative for individuals with mTORopathies, where current treatments often rely on symptom management rather than addressing the underlying genetic cause.

## 5. Current Advances in mTOR Gene Therapies

The treatment of pathologies associated with the mechanistic target of rapamycin (mTOR) remains challenging, primarily due to the limited efficacy of rapamycin [113,114,115], the standard inhibitor used for this pathway. Additionally, the inhibition of mTOR is complex because its activity depends on the formation of distinct protein complexes—mTORC1, mTORC2, and mTORC3—with each playing unique roles in cellular processes and responding differently to rapamycin. This complexity, coupled with mTOR’s critical involvement in various diseases such as cancer and epilepsy, underscores the need for alternative therapeutic approaches, including gene therapy.

Focal cortical dysplasia (FCD) is one of the primary causes of refractory epilepsy in children, with disruptions in the mTOR pathway playing a significant role in the epileptogenesis of these disorders. Recently, Barbanoj et al. (2024) [116] developed a viral vector therapy aimed at treating FCD. The researchers utilized a mouse model where they overexpressed the Ras homolog enriched in brain (RHEB), an mTORC1 activator, to mimic FCD. Using in utero electroporation, they introduced an RHEB plasmid into frontal lobe neural progenitors. The therapeutic intervention involved injecting a modified adeno-associated viral vector (AAV9) carrying an engineered potassium channel (EKC) transgene, which effectively reduced the seizure frequency by 64% without directly targeting an mTOR mutation.

Another promising approach is highlighted by Lee et al. (2021) [117] who designed an adeno-associated viral therapy (rAAV2-shmTOR-SD) to inhibit mTOR in mouse models of diabetic retinopathy (DR). By delivering a short-hairpin RNA (shRNA) to reduce mTOR expression, they observed a reduction in DR-related symptoms, including the preservation of retinal structure and decreased vascular leakage. Cha et al. (2022) [118] further demonstrated in an in vitro model that this therapy could inhibit neovascularization by blocking VEGF, a process regulated by mTOR [119].

As gene-editing technologies like CRISPR/Cas9 and Prime Editing continue to evolve, genetic therapies targeting mTOR-related conditions may soon become more common. However, while RNA-based therapies provide temporary effects due to the transient nature of RNA, DNA-editing therapies offer the potential for permanent genetic modifications. Both strategies face the significant challenge of delivery efficiency—how many cells can be reached and successfully modified? Achieving effective transfection, even in controlled in vitro environments, remains difficult, and scaling this up to the human body poses additional hurdles. Ultimately, the most effective genetic therapy will be one that provides long-lasting effects with minimal side effects.

## 6. Vectors for Gene-Editing Tool Delivery: Innovations in Precision and Targeted Therapeutics

**Figure 2:**
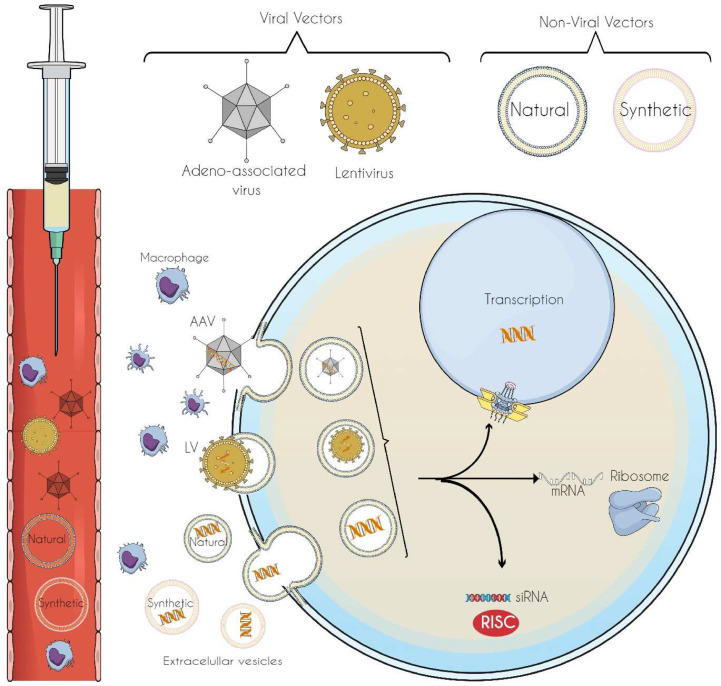
Viral and non-viral vectors can be employed to deliver siRNAs, microRNAs, and drugs to cells. Once in the bloodstream, these vectors extravasate into the extracellular matrix and undergo endocytosis. Inside the cell, the vectors may transport their cargo to the nucleus or ribosomes or simply release the siRNA.

Gene delivery vectors (Figure 2), whether viral or non-viral, are engineered to transport genetic materials such as genes, exons, or gene expression modulators—to specific regions in the body to enable the modulation of protein production essential for various cellular functions. These vectors, paired with technologies like CRISPR-Cas9, allow for targeted gene silencing, activation, or modification in specific organs, such as the central nervous system (CNS), ensuring precise delivery of the therapeutic material.

### 6.1. Viral Vectors

Viral vectors are derived from non-pathogenic viruses. The most studied vectors are adeno-associated viruses (AAVs) and lentiviruses [120,121]. AAVs can package around ~4.7 kb of DNA [122], whereas lentiviruses can carry larger amounts of genetic material, approximately ~9.8 kb [123]. However, lentiviruses are significantly larger (~100 nm compared with AAV’s ~20 nm), which can hinder their diffusion through the extracellular matrix, limiting their effectiveness in reaching target tissues [124].

A key challenge with AAV vectors is the presence of antibodies in humans against adenoviruses, which are naturally present in the microbiota. To overcome this, studies have explored AAV capsids derived from non-human vertebrates, such as porcine sources. These capsids evade neutralization by human IgG antibodies and have the ability to cross the blood–brain barrier (BBB) [120]. Hybrid AAVs, which are recombinantly engineered, are also being developed to improve immune evasion and vector delivery [120]. The production of these recombinant AAVs (rAAVs) is typically done using HEK293 cells [125], which are co-transfected with plasmids containing viral proteins and genomes. A notable advantage of lentiviruses is that, unlike AAVs, they generally do not elicit significant immune responses [124].

Studies show that different AAV serotypes can effectively modulate gene expression in specific tissues under pathological conditions. For instance, one study used AAV1 to deliver neuropeptide Y (NPY) and its receptor (Y2) to the CNS of rats with induced seizures [126]. This treatment increased the seizure latency and reduced the seizure duration, highlighting AAV’s potential in treating epilepsy. Another example involves the use of AAV9 to enhance the expression of the SCN1A gene [122] in a model of Dravet syndrome, a refractory epilepsy caused by heterozygous loss-of-function mutations in the gene [127,128]. This treatment increased sodium channel expression and significantly reduced the seizure frequency, with 68% of the treated animals becoming seizure-free.

In the context of mTORopathies, viral vectors can be utilized to replace dysfunctional proteins. For example, in a study involving a model of tuberous sclerosis complex 1 (TSC1) [83], AAVs were used to replace hamartin, a protein critical for mTOR regulation. The treatment with AAV9-hamartin significantly extended survival in the animal model, demonstrating the efficacy of viral vectors in studying and treating diseases involving the mTOR pathway.

In understanding the biological mechanisms behind seizure manifestation, researchers [129] have explored the role of LRP4, a protein primarily expressed at neuromuscular junctions that shows altered expression during seizure events. In a key study, silencing LRP4 in rodent models of pilocarpine-induced epilepsy using lentiviral vectors demonstrated a neuroprotective effect. Injection of a lentivirus containing an RNA silencer targeting LRP4 transcription into the hippocampal molecular layer led to increased seizure latency, meaning that higher doses of pilocarpine were required to induce seizures in the treated animals. These findings suggest that LRP4 silencing could offer neuroprotection and contribute to the development of novel therapeutic strategies for controlling seizures, especially in pharmacologically resistant cases such as those seen in mTORopathies. This line of research supports the potential of gene therapy, particularly the delivery of lentiviral vectors, as an alternative approach to treating epilepsy, which is refractory to conventional treatments.

Recent research has increasingly focused on gene therapies as a viable strategy for treating neurodegenerative diseases. A notable study by Ng et al. (2021) [130] investigated induced pluripotent stem cells (iPSCs) derived from patients with a homozygous loss-of-function mutation in the SLC6A3 gene, which encodes the dopamine transporter responsible for the reuptake of dopamine in presynaptic dopaminergic neurons. The absence of this transporter can lead to symptoms resembling Parkinson’s disease. The researchers demonstrated that by introducing a lentivirus carrying the complementary DNA of SLC6A3, it was possible to improve dopamine reuptake, effectively halting neurodegeneration, even in the presence of ongoing dysregulation of the MAO-A and MAO-B enzymes. Furthermore, the team delivered AAV2 containing the SLC6A3 gene directly into the substantia nigra, specifically targeting the dopamine-deficient region. Remarkably, the group receiving the highest viral dose (2 × 10^10^ vg) exhibited motor behaviors indistinguishable from those of the non-mutated control group, even after 8 weeks. These findings not only underscore the potential of both lentiviruses and AAVs in delivering gene therapies but also highlight their role in addressing secondary conditions like neurodegeneration in patients with mutations affecting the mTOR pathway. Such advancements could pave the way for new therapeutic avenues in managing neurodegenerative disorders associated with dysfunctions in dopamine signaling and mTOR regulation.

Despite the promising potential of adenoviruses and lentiviruses as vectors for delivering gene therapies, there remain relatively few clinical trials focused on central nervous system (CNS) diseases utilizing these systems (Table 2). Moreover, there are no suspended trials at present; however, it is important to note that detailed information regarding trial suspensions is not always publicly disclosed. In contrast, oncology—a field frequently impacted by dysregulation of the mTOR pathway in various cancers [131]—has witnessed numerous successful trials employing viral vectors. These successes highlight the effectiveness of such vectors in targeting cancerous cells and managing tumor growth [132,133,134]. While the application of viral vectors in CNS diseases is still evolving, their established efficacy in oncology underscores their potential as a viable therapeutic option for gene delivery.

However, the treatment of CNS diseases with viral vectors faces significant challenges, with the blood–brain barrier (BBB) being a major obstacle as it restricts vector penetration into the brain circulatory system [141]. Additionally, immune responses against the vector can compromise its efficacy, particularly with repeated administrations [142]. The distribution of viral vectors within the CNS is also limited due to the dense extracellular matrix, hindering diffusion and restricting transgene expression to localized regions [143]. On the other hand, in tumor models, viral vectors offer advantages such as selective tropism for neoplastic cells, the ability to deliver pro-apoptotic genes, and their application in oncolytic therapies due to their ability to promote tumor destruction and to activate an immune response against cancer [144,145]. An alternative to mitigate these limitations is the use of non-viral vectors.

### 6.2. Non-Viral Vectors

Nanomedicine-based non-viral vectors, whether synthetic or natural, represent a promising advancement in precision medicine, providing a targeted and efficient means for gene editing and modification [146,147,148,149]. In the realm of modern medicine, nanomedicine has already made significant strides, utilizing synthetic non-viral vectors such as polymers, liposomes, lipid carriers, and inorganic nanomaterials for the diagnosis, prevention, and treatment of a diverse array of diseases in clinical practice [150,151]. Additionally, there remains substantial potential for growth in this area through the exploration of natural non-viral vectors, such as extracellular vesicles (e.g., exosomes), which show promise as therapeutic delivery systems in preclinical settings [152,153,154,155]. This shift could greatly advance the field of gene therapy [149].

The application of non-viral vectors, particularly synthetic nanoparticles, has attracted considerable attention in both preclinical and clinical environments due to their ability to enhance drug delivery and therapeutic effectiveness [156,157]. Nonetheless, concerns about their safety and long-term impacts continue to pose challenges [158]. Preclinical research indicates that nanoparticles can effectively target specific tissues while minimizing off-target effects. However, issues surrounding their biocompatibility, biodistribution, and clearance mechanisms necessitate further investigation to ensure their safe clinical applications [159,160]. Although some nanoparticle-based therapies have received clinical approval, hurdles remain, including the potential toxicity, immune responses, and risks of nanoparticle accumulation in critical organs, which may impede their broader implementation [161].

Over the last three decades, significant advancements have been made in developing synthetic nanoparticle-based non-viral vectors aimed at overcoming the limitations of traditional treatments for various conditions [150,161]. These vectors are engineered to reduce side effects and toxicity while enhancing therapeutic precision, stability, solubility, biodistribution, and controlled drug release [162,163]. Their ability to address these limitations broadens their potential applications across multiple medical domains.

In oncology, nanoparticles have had a profound impact, effectively addressing key challenges such as therapy resistance, limited therapeutic effects on target tissues, and collateral damage to healthy tissues [164,165]. Despite these advancements, much of the nanoparticle research remains in preclinical phases due to the need for thorough safety and efficacy validation before transitioning to clinical applications [166,167]. Nonetheless, a select few nanomedicines have advanced to clinical trials, highlighting both the potential and challenges that persist in the development of nanoparticle-based therapies (Table 3).

Synthetic non-viral vectors have emerged as a significant tool in clinical medicine, facilitating the treatment of various diseases, including COVID-19, cancer, schizophrenia, and multiple sclerosis [163,168,172,176], and their ability to deliver molecular cargo, such as nucleoside-modified messenger RNA (modRNA), has shown considerable therapeutic potential [169]. However, gene therapy poses unique challenges, necessitating vectors with highly specific recognition capabilities to accurately target cells within particular tissues—an ability that current synthetic vector technologies still struggle to fully achieve [178,179,180]. In this context, natural non-viral vectors, such as, are emerging as promising candidates for localized gene therapy, offering enhanced biocompatibility and target specificity [181,182]. These natural vectors may help overcome the limitations associated with synthetic vectors and offer safer delivery of genetic material to diseased tissues.

When selecting the most appropriate non-viral vector for a specific therapeutic application, several key parameters must be considered [183]. The route of administration is critical; for instance, vectors delivered via intravenous injection must navigate challenges such as rapid clearance from the bloodstream and immune recognition, while localized delivery requires vectors with high tissue specificity [156,184,185]. Also, in pathologies such as mTORopathies, it is important to ensure the use of vectors that cross the blood–brain barrier, a characteristic naturally found in extracellular vesicles (EVs) [186]. The pathology of the target disease also plays a significant role, as different tissues and cell types have unique characteristics that influence the vector’s efficiency in reaching and penetrating target cells [187]. Additionally, the desired therapeutic effect—be it increased circulation time, enhanced drug stability, or precise targeting of specific tissues—will dictate the vector design [85,188,189]. These parameters must be meticulously optimized to ensure that the chosen vector delivers the therapeutic payload effectively while minimizing off-target effects and toxicity.

Non-viral vectors offer several advantages that enhance their reliability as drug delivery systems [161]. Synthetic vectors, including lipid nanoparticles and polymer-based carriers, can be engineered with tunable properties such as size, surface charge, and biodegradability, making them versatile across a range of applications [161]. Natural non-viral vectors, such as extracellular vesicles and exosomes, provide high biocompatibility and can evade immune detection due to their endogenous origin [154,155,161]. Both types of vectors exhibit low immunogenicity compared with viral vectors, making them safer for repeated administration. Moreover, non-viral vectors can accommodate large genetic payloads, including plasmids, RNA, small molecules, and even gene therapy components like CRISPR/Cas9, which are critical for the success of gene therapy and targeted treatments.

Despite existing technological limitations in the design and engineering of non-viral vectors [85], significant advancements have been made over the last few decades [161]. Progress in nanotechnology, molecular biology, and materials science has led to the development of more sophisticated delivery systems with improved targeting, stability, and efficiency. These ongoing advancements highlight the potential of non-viral vectors as vehicles for gene therapy as these systems continue to evolve. While challenges persist in enhancing tissue specificity and payload capacity, the progress achieved thus far underscores the promising role that non-viral vectors are poised to play in the future of personalized medicine and targeted therapies.

## 7. The Future of Gene Editing for mTORopathies

Advances in gene-editing technology, particularly for mTORopathies, are poised to revolutionize the treatment landscape for these complex disorders. mTORopathies, characterized by dysregulation of the mTOR signaling pathway, present unique challenges due to the pathway’s integral roles in cell growth, proliferation, and metabolism [43,190,191]. As tools like CRISPR-Cas9 evolve [190], their precision in modulating mTOR-related pathways becomes increasingly feasible, potentially allowing for tailored interventions that target the specific genetic mutations underlying these conditions [192]. While CRISPR-Cas9-based treatments for mTORopathies are not yet available, this technology has been instrumental in elucidating the molecular mechanisms underlying these disorders. Recent genome-wide screenings have identified key regulators of the mTORC1 pathway, highlighting the role of mitochondrial function and cellular stress in its activation. Additionally, studies have demonstrated the significance of mTORC1 in glucocorticoid sensitivity in leukemia cells, revealing potential therapeutic targets [193,194]. These advances underscore the potential of CRISPR-Cas9 in deepening our understanding of mTORopathies and guiding the development of future therapeutic strategies.

One of the most promising avenues for advancing treatment is the development of targeted delivery mechanisms, such as non-viral vectors and nanotechnology. These innovations can enhance the specificity of gene-editing therapies, minimizing off-target effects and ensuring that therapeutic interventions are efficiently delivered to affected cells. This is especially important given the diverse phenotypic manifestations associated with mTORopathies, which range from neurological symptoms to metabolic dysregulation [192,195]. By addressing the full spectrum of these phenotypes, gene-editing technologies could significantly improve patient outcomes.

However, alongside the potential benefits of these technological advancements are significant ethical and regulatory challenges. The complexity of mTOR signaling raises concerns about unintended consequences from gene editing, such as off-target effects that could disrupt other critical cellular processes. The long-term implications of editing such a central regulatory pathway remain largely unknown, necessitating rigorous preclinical testing and ongoing monitoring [191].

Furthermore, the ethical debate intensifies in the context of germline modifications, which could impact future generations. Regulatory frameworks must evolve to keep pace with these advances, ensuring safety, equity, and access to these technologies while addressing their broader societal implications [196,197]. Establishing clear guidelines and ethical standards will be essential as gene editing for mTORopathies moves closer to clinical application, ensuring that the promise of these technologies is realized responsibly and effectively.

## 8. Conclusions

mTORopathies, characterized by dysregulation of the mTOR signaling pathway, have emerged as a critical focus in the study of neurodevelopmental disorders and refractory epilepsy. The strong connection between hyperactivation of this pathway and conditions such as TSC, FCD2, HME, SKS, POLR2A-related syndrome associated with epilepsy and mental retardation, PMSE, PTEN syndrome, DEPDC5-related syndrome, and PROS underscores the central role of mTOR in neurodevelopmental and epileptogenic processes. Current therapeutic strategies, particularly the use of mTOR inhibitors, have demonstrated significant efficacy in controlling tumor growth, reducing seizure frequency, and improving the quality of life for affected individuals.

However, the limitations of these treatments in addressing the underlying genetic mutations necessitate more targeted approaches. The rapid advancements in gene-editing technologies, notably CRISPR/Cas9, offer a promising avenue for precision therapies aimed at correcting the genetic abnormalities that drive mTORopathies. While gene therapies hold transformative potential, they also present challenges related to delivery specificity, long-term safety, and ethical considerations, particularly in the context of germline editing.

Recent advances in nanomedicine have underscored the potential of non-viral vectors in gene therapy. Among these, extracellular vesicles (EVs) have garnered significant interest due to their natural origin, excellent biocompatibility, and inherent ability to traverse the blood–brain barrier. EVs, including exosomes, offer targeted delivery of gene-editing tools with minimal immunogenicity and a reduced risk of off-target effects. Their unique properties position them as a promising platform for precision therapies aimed at correcting the genetic mutations underlying mTORopathies. This evolving landscape represents a transformative frontier in neurodevelopmental and neurological therapeutics, with the promise of more personalized and effective treatment strategies for patients affected by mTORopathies.

Looking forward, continued research into the molecular mechanisms of mTORopathies and the development of innovative gene therapies will be pivotal in shifting the paradigm from symptomatic treatment to curative interventions. This evolving field stands at the intersection of molecular genetics, neurology, and therapeutic innovation, offering hope for more effective and personalized treatments for these complex disorders.

## Figures and Tables

**Table 2:** Clinically approved treatments with viral vectors for CNS diseases (2000–2024).

Approval Year	Virus/Serotype	Clinical Application	Drug/Cargo Type	Reference
2000	pAAV	Canavan disease	Human aspartoacylase cDNA	[135]
2004	AAV2	Late infantile neuronal ceroid lipofuscinosis	human CLN2 cDNA	[136]
2006	AAV2	Canavan disease	Human aspartoacylase cDNA	[137]
2008	AAV2	Late infantile neuronal ceroid lipofuscinosis	Human aspartoacylase cDNA	[138]
2014	Lentivirus	neuroAIDS	human (h)BDNF	[139]
2016	Lentivirus	Metachromatic leukodystrophy	Human ARSA cDNA	[140]

**Table 3:** Clinically approved nanomedicine-based non-viral vectors (2011–2021).

Approval Year	Nanoparticle Type	Clinical Application	Drug/Cargo Type	Reference
2021	Liposome	COVID-19	mRNA-1273 Nucleoside-modified RNA (modRNA)	[168,169]
2019	Metal nanoparticle	Advanced soft-tissue sarcoma	Hafnium oxide	[170]
2018	Liposome	Polyneuropathy caused by hereditary ATTR amyloidosis	siRNA	[171]
2018	Nanocrystals	Schizophrenia	Aripiprazole lauroxil	[172]
2017	Liposome	Acute myeloid leukemia	Daunorubicin and cytarabine	[173]
2015	Liposome	Metastatic pancreatic cancer	Irinotecan chemotherapy	[174]
2015	Polymeric nanoparticle	Ovarian cancer	Paclitaxel chemotherapy	[175]
2014	Polymeric nanoparticle	Multiple sclerosis	Polymer–protein conjugate (PEGylated IFN beta-1a)	[163]
2013	Polymeric nanoparticle	Crohn’s disease; Rheumatoid arthritis; Psoriatic arthritis; Ankylosing spondylitis	PEGylated antibody fragment (Certolizumab)	[163]
2013	Protein nanoparticles	Breast cancer; Non-small-cell lung cancer; Pancreatic cancer	Albumin-bound paclitaxel chemotherapy	[176]
2011	Liposome	Bupivacaine	Pain treatment	[177]

## Data Availability

No new data were created or analyzed in this study. Data sharing is not applicable to this article.

## References

[1] Wipperman M.F., Montrose D.C., Gotto A.M., Hajjar D.P. (2019). Mammalian Target of Rapamycin: A Metabolic Rheostat for Regulating Adipose Tissue Function and Cardiovascular Health. Am. J. Pathol..

[2] Committee H.G.N. MTOR (Mechanistic Target Of Rapamycin Kinase). https://www.genenames.org/data/gene-symbol-report/#!/hgnc_id/HGNC:3942.

[3] Panwar V., Singh A., Bhatt M., Tonk R.K., Azizov S., Raza A.S., Sengupta S., Kumar D., Garg M. (2023). Multifaceted role of mTOR (mammalian target of rapamycin) signaling pathway in human health and disease. Signal Transduct. Target Ther..

[4] Vezina C., Kudelski A., Sehgal S.N. (1975). Rapamycin (AY-22,989), a new antifungal antibiotic. I. Taxonomy of the producing streptomycete and isolation of the active principle. J. Antibiot..

[5] el Hage A., Dormond O. (2021). Combining mTOR Inhibitors and T Cell-Based Immunotherapies in Cancer Treatment. Cancers.

[6] Ebner M., Sinkovics B., Szczygiel M., Ribeiro D.W., Yudushkin I. (2017). Localization of mTORC2 activity inside cells. J. Cell Biol..

[7] Szwed A., Kim E., Jacinto E. (2021). Regulation and metabolic functions of mTORC1 and mTORC2. Physiol. Rev..

[8] Nicklin P., Bergman P., Zhang B., Triantafellow E., Wang H., Nyfeler B., Yang H., Hild M., Kung C., Wilson C. (2009). Bidirectional transport of amino acids regulates mTOR and autophagy. Cell.

[9] Kim E., Goraksha-Hicks P., Li L., Neufeld T.P., Guan K.L. (2008). Regulation of TORC1 by Rag GTPases in nutrient response. Nat. Cell Biol..

[10] Jacinto E., Loewith R., Schmidt A., Lin S., Ruegg M.A., Hall A., Hall M.N. (2004). Mammalian TOR complex 2 controls the actin cytoskeleton and is rapamycin insensitive. Nat. Cell Biol..

[11] Sarbassov D.D., Guertin D.A., Ali S.M., Sabatini D.M. (2005). Phosphorylation and regulation of Akt/PKB by the rictor-mTOR complex. Science.

[12] Saxton R.A., Sabatini D.M. (2017). mTOR Signaling in Growth, Metabolism, and Disease. Cell.

[13] Guri Y., Colombi M., Dazert E., Hindupur S.K., Roszik J., Moes S., Jenoe P., Heim M.H., Riezman I., Riezman H. (2017). mTORC2 Promotes Tumorigenesis via Lipid Synthesis. Cancer Cell.

[14] Liu G.Y., Sabatini D.M. (2020). mTOR at the nexus of nutrition, growth, ageing and disease. Nat. Rev. Mol. Cell Biol..

[15] Driedger J.H., Schroter J., Group P.R.-S., Syrbe S., Saffari A. (2025). Long-term neuropsychologic outcome of pre-emptive mTOR inhibitor treatment in children with tuberous sclerosis complex (TSC) under 4 months of age (PROTECT), a two-arm, randomized, observer-blind, controlled phase IIb national multicentre clinical trial: Study protocol. Orphanet J. Rare Dis..

[16] Ryskalin L., Limanaqi F., Frati A., Busceti C.L., Fornai F. (2018). mTOR-Related Brain Dysfunctions in Neuropsychiatric Disorders. Int. J. Mol. Sci..

[17] Koike-Kumagai M., Fujimoto M., Wataya-Kaneda M. (2022). Sirolimus relieves seizures and neuropsychiatric symptoms via changes of microglial polarity in tuberous sclerosis complex model mice. Neuropharmacology.

[18] Cui F., Gu S., Gu Y., Yin J., Fang C., Liu L. (2021). Alteration in the mRNA expression profile of the autophagy-related mTOR pathway in schizophrenia patients treated with olanzapine. BMC Psychiatry.

[19] Nawwar D.A., Zaki H.F., Sayed R.H. (2022). Role of the NRG1/ErbB4 and PI3K/AKT/mTOR signaling pathways in the anti-psychotic effects of aripiprazole and sertindole in ketamine-induced schizophrenia-like behaviors in rats. Inflammopharmacology.

[20] Hoeffer C.A., Klann E. (2010). mTOR signaling: At the crossroads of plasticity, memory and disease. Trends Neurosci..

[21] Tang S.J., Reis G., Kang H., Gingras A.C., Sonenberg N., Schuman E.M. (2002). A rapamycin-sensitive signaling pathway contributes to long-term synaptic plasticity in the hippocampus. Proc. Natl. Acad. Sci. USA.

[22] Lipton J.O., Sahin M. (2014). The neurology of mTOR. Neuron.

[23] Luo Y., Liu L., Wu Y., Singh K., Su B., Zhang N., Liu X., Shen Y., Huang S. (2015). Rapamycin inhibits mSin1 phosphorylation independently of mTORC1 and mTORC2. Oncotarget.

[24] Harwood F.C., Klein Geltink R.I., O’Hara B.P., Cardone M., Janke L., Finkelstein D., Entin I., Paul L., Houghton P.J., Grosveld G.C. (2018). ETV7 is an essential component of a rapamycin-insensitive mTOR complex in cancer. Sci. Adv..

[25] Zhan J., Harwood F., Have S.T., Lamond A., Phillips A.H., Kriwacki R.W., Halder P., Cardone M., Grosveld G.C. (2024). Assembly of mTORC3 Involves Binding of ETV7 to Two Separate Sequences in the mTOR Kinase Domain. Int. J. Mol. Sci..

[26] Takei N., Inamura N., Kawamura M., Namba H., Hara K., Yonezawa K., Nawa H. (2004). Brain-derived neurotrophic factor induces mammalian target of rapamycin-dependent local activation of translation machinery and protein synthesis in neuronal dendrites. J. Neurosci..

[27] Lenz G., Avruch J. (2005). Glutamatergic regulation of the p70S6 kinase in primary mouse neurons. J. Biol. Chem..

[28] Feng Z., Levine A.J. (2010). The regulation of energy metabolism and the IGF-1/mTOR pathways by the p53 protein. Trends Cell Biol.

[29] Zhao W., Xie C., Zhang X., Liu J., Liu J., Xia Z. (2023). Advances in the mTOR signaling pathway and its inhibitor rapamycin in epilepsy. Brain Behav..

[30] Zhang X., Wu Y., Sun X., Cui Q., Bai X., Dong G., Gao Z., Wang Y., Gao C., Sun S. (2022). The PI3K/AKT/mTOR signaling pathway is aberrantly activated in primary central nervous system lymphoma and correlated with a poor prognosis. BMC Cancer.

[31] Kwon C.H., Luikart B.W., Powell C.M., Zhou J., Matheny S.A., Zhang W., Li Y., Baker S.J., Parada L.F. (2006). Pten regulates neuronal arborization and social interaction in mice. Neuron.

[32] Chen C.J., Sgritta M., Mays J., Zhou H., Lucero R., Park J., Wang I.C., Park J.H., Kaipparettu B.A., Stoica L. (2019). Therapeutic inhibition of mTORC2 rescues the behavioral and neurophysiological abnormalities associated with Pten-deficiency. Nat. Med..

[33] Ma Q., Chen G., Li Y., Guo Z., Zhang X. (2024). The molecular genetics of PI3K/PTEN/AKT/mTOR pathway in the malformations of cortical development. Genes Dis..

[34] Girodengo M., Ultanir S.K., Bateman J.M. (2022). Mechanistic target of rapamycin signaling in human nervous system development and disease. Front. Mol. Neurosci..

[35] Luo C., Ye W.R., Shi W., Yin P., Chen C., He Y.B., Chen M.F., Zu X.B., Cai Y. (2022). Perfect match: mTOR inhibitors and tuberous sclerosis complex. Orphanet J. Rare Dis..

[36] Gerasimenko A., Baldassari S., Baulac S. (2023). mTOR pathway: Insights into an established pathway for brain mosaicism in epilepsy. Neurobiol. Dis..

[37] Nguyen L.H., Bordey A. (2021). Convergent and Divergent Mechanisms of Epileptogenesis in mTORopathies. Front. Neuroanat..

[38] Liu A.C., Shen Y., Serbinski C.R., He H., Roman D., Endale M., Aschbacher-Smith L., King K.A., Granadillo J.L., Lopez I. (2024). Clinical and functional studies of MTOR variants in Smith-Kingsmore syndrome reveal deficits of circadian rhythm and sleep-wake behavior. HGG Adv..

[39] Besterman A.D., Althoff T., Elfferich P., Gutierrez-Mejia I., Sadik J., Bernstein J.A., van Ierland Y., Kattentidt-Mouravieva A.A., Nellist M., Abramson J. (2021). Functional and structural analyses of novel Smith-Kingsmore Syndrome-Associated MTOR variants reveal potential new mechanisms and predictors of pathogenicity. PLoS Genet..

[40] Poole R.L., Curry P.D.K., Marcinkute R., Brewer C., Coman D., Hobson E., Johnson D., Lynch S.A., Saggar A., Searle C. (2021). Delineating the Smith-Kingsmore syndrome phenotype: Investigation of 16 patients with the MTOR c.5395G > A p.(Glu1799Lys) missense variant. Am. J. Med. Genet. A.

[41] Gordo G., Tenorio J., Arias P., Santos-Simarro F., Garcia-Minaur S., Moreno J.C., Nevado J., Vallespin E., Rodriguez-Laguna L., de Mena R. (2018). mTOR mutations in Smith-Kingsmore syndrome: Four additional patients and a review. Clin. Genet..

[42] Baldassari S., Ribierre T., Marsan E., Adle-Biassette H., Ferrand-Sorbets S., Bulteau C., Dorison N., Fohlen M., Polivka M., Weckhuysen S. (2019). Dissecting the genetic basis of focal cortical dysplasia: A large cohort study. Acta Neuropathol..

[43] Auvin S., Baulac S. (2023). mTOR-therapy and targeted treatment opportunities in mTOR-related epilepsies associated with cortical malformations. Rev. Neurol..

[44] Lee W.S., Baldassari S., Stephenson S.E.M., Lockhart P.J., Baulac S., Leventer R.J. (2022). Cortical Dysplasia and the mTOR Pathway: How the Study of Human Brain Tissue Has Led to Insights into Epileptogenesis. Int. J. Mol. Sci..

[45] Di Rocco C., Battaglia D., Pietrini D., Piastra M., Massimi L. (2006). Hemimegalencephaly: Clinical implications and surgical treatment. Childs Nerv. Syst..

[46] Garcia C.A.B., Carvalho S.C.S., Yang X., Ball L.L., George R.D., James K.N., Stanley V., Breuss M.W., Thome U., Santos M.V. (2020). mTOR pathway somatic variants and the molecular pathogenesis of hemimegalencephaly. Epilepsia Open.

[47] Mirzaa G.M., Poduri A. (2014). Megalencephaly and hemimegalencephaly: Breakthroughs in molecular etiology. Am. J. Med. Genet. C Semin. Med. Genet..

[48] Sidira C., Vargiami E., Dragoumi P., Zafeiriou D.I. (2021). Hemimegalencephaly and tuberous sclerosis complex: A rare yet challenging association. Eur. J. Paediatr. Neurol..

[49] Venot Q., Canaud G. (2017). [PIK3CA-related overgrowth syndrome (PROS)]. Nephrol. Ther..

[50] Keppler-Noreuil K.M., Rios J.J., Parker V.E., Semple R.K., Lindhurst M.J., Sapp J.C., Alomari A., Ezaki M., Dobyns W., Biesecker L.G. (2015). PIK3CA-related overgrowth spectrum (PROS): Diagnostic and testing eligibility criteria, differential diagnosis, and evaluation. Am. J. Med. Genet. A.

[51] Dang L.T., Vaid S., Lin G., Swaminathan P., Safran J., Loughman A., Lee M., Glenn T., Majolo F., Crino P.B. (2021). STRADA-mutant human cortical organoids model megalencephaly and exhibit delayed neuronal differentiation. Dev. Neurobiol..

[52] Galanopoulou A.S., Gorter J.A., Cepeda C. (2012). Finding a better drug for epilepsy: The mTOR pathway as an antiepileptogenic target. Epilepsia.

[53] Puffenberger E.G., Strauss K.A., Ramsey K.E., Craig D.W., Stephan D.A., Robinson D.L., Hendrickson C.L., Gottlieb S., Ramsay D.A., Siu V.M. (2007). Polyhydramnios, megalencephaly and symptomatic epilepsy caused by a homozygous 7-kilobase deletion in LYK5. Brain.

[54] Orlova K.A., Parker W.E., Heuer G.G., Tsai V., Yoon J., Baybis M., Fenning R.S., Strauss K., Crino P.B. (2010). STRADalpha deficiency results in aberrant mTORC1 signaling during corticogenesis in humans and mice. J. Clin. Investig..

[55] Haijes H.A., Koster M.J.E., Rehmann H., Li D., Hakonarson H., Cappuccio G., Hancarova M., Lehalle D., Reardon W., Schaefer G.B. (2019). De Novo Heterozygous POLR2A Variants Cause a Neurodevelopmental Syndrome with Profound Infantile-Onset Hypotonia. Am. J. Hum. Genet..

[56] Giacomini T., Scala M., Nobile G., Severino M., Tortora D., Nobili L., Accogli A., Torella A., Capra V., Mancardi M.M. (2022). De novo POLR2A p.(Ile457Thr) variant associated with early-onset encephalopathy and cerebellar atrophy: Expanding the phenotypic spectrum. Brain Dev..

[57] Zhang Y.Z., Zeb A., Cheng L.F. (2022). Exploring the molecular mechanism of hepatitis virus inducing hepatocellular carcinoma by microarray data and immune infiltrates analysis. Front. Immunol..

[58] Kreis P., Leondaritis G., Lieberam I., Eickholt B.J. (2014). Subcellular targeting and dynamic regulation of PTEN: Implications for neuronal cells and neurological disorders. Front. Mol. Neurosci..

[59] Cullen E.R., Safari M., Mittelstadt I., Weston M.C. (2024). Hyperactivity of mTORC1- and mTORC2-dependent signaling mediates epilepsy downstream of somatic PTEN loss. Elife.

[60] Tariq K., Cullen E., Getz S.A., Conching A.K.S., Goyette A.R., Prina M.L., Wang W., Li M., Weston M.C., Luikart B.W. (2022). Disruption of mTORC1 rescues neuronal overgrowth and synapse function dysregulated by Pten loss. Cell Rep..

[61] Baulac S., Baldassari S., Adam M.P., Feldman J., Mirzaa G.M., Pagon R.A., Wallace S.E., Amemiya A. (1993). DEPDC5-Related Epilepsy. GeneReviews(^®^).

[62] Samanta D. (2022). DEPDC5-related epilepsy: A comprehensive review. Epilepsy Behav..

[63] Yuskaitis C.J., Jones B.M., Wolfson R.L., Super C.E., Dhamne S.C., Rotenberg A., Sabatini D.M., Sahin M., Poduri A. (2018). A mouse model of DEPDC5-related epilepsy: Neuronal loss of Depdc5 causes dysplastic and ectopic neurons, increased mTOR signaling, and seizure susceptibility. Neurobiol. Dis..

[64] Doudna J.A., Charpentier E. (2014). Genome editing. The new frontier of genome engineering with CRISPR-Cas9. Science.

[65] Adashi E.Y., Gruppuso P.A., Cohen I.G. (2024). CRISPR Therapy of Sickle Cell Disease: The Dawning of the Gene Editing Era. Am. J. Med..

[66] Frangoul H., Altshuler D., Cappellini M.D., Chen Y.S., Domm J., Eustace B.K., Foell J., de la Fuente J., Grupp S., Handgretinger R. (2021). CRISPR-Cas9 Gene Editing for Sickle Cell Disease and beta-Thalassemia. N. Engl. J. Med..

[67] Pauling L., Itano H.A., Singer S.J., Wells I.C. (1949). Sickle cell anemia a molecular disease. Science.

[68] Avery O.T., Macleod C.M., McCarty M. (1944). Studies on the Chemical Nature of the Substance Inducing Transformation of Pneumococcal Types: Induction of Transformation by a Desoxyribonucleic Acid Fraction Isolated from Pneumococcus Type Iii. J. Exp. Med..

[69] Watson J.D., Crick F.H. (2007). Molecular structure of nucleic acids: A structure for deoxyribose nucleic acid. Clin. Orthop. Relat. Res..

[70] Rogers S., Lowenthal A., Terheggen H.G., Columbo J.P. (1973). Induction of arginase activity with the Shope papilloma virus in tissue culture cells from an argininemic patient. J. Exp. Med..

[71] Terheggen H.G., Lowenthal A., Lavinha F., Colombo J.P., Rogers S. (1975). Unsuccessful trial of gene replacement in arginase deficiency. Z. Kinderheilkd.

[72] Beutler E. (2001). The Cline affair. Mol. Ther..

[73] Rosenberg S.A., Anderson W.F., Blaese M., Hwu P., Yannelli J.R., Yang J.C., Topalian S.L., Schwartzentruber D.J., Weber J.S., Ettinghausen S.E. (1993). The development of gene therapy for the treatment of cancer. Ann. Surg..

[74] Wirth T., Parker N., Yla-Herttuala S. (2013). History of gene therapy. Gene.

[75] Advanced Therapy Medicinal Products: Overview. https://www.ema.europa.eu/en/human-regulatory-overview/advanced-therapy-medicinal-products-overview.

[76] https://www.fda.gov/.

[77] Blair J.D., Bateup H.S. (2020). New frontiers in modeling tuberous sclerosis with human stem cell-derived neurons and brain organoids. Dev. Dyn..

[78] Parker W.E., Orlova K.A., Parker W.H., Birnbaum J.F., Krymskaya V.P., Goncharov D.A., Baybis M., Helfferich J., Okochi K., Strauss K.A. (2013). Rapamycin prevents seizures after depletion of STRADA in a rare neurodevelopmental disorder. Sci. Transl. Med..

[79] Marsan E., Ishida S., Schramm A., Weckhuysen S., Muraca G., Lecas S., Liang N., Treins C., Pende M., Roussel D. (2016). Depdc5 knockout rat: A novel model of mTORopathy. Neurobiol. Dis..

[80] Cupolillo D., Hoxha E., Faralli A., De Luca A., Rossi F., Tempia F., Carulli D. (2016). Autistic-Like Traits and Cerebellar Dysfunction in Purkinje Cell PTEN Knock-Out Mice. Neuropsychopharmacology.

[81] Niu W., Siciliano B., Wen Z. (2024). Modeling tuberous sclerosis complex with human induced pluripotent stem cells. World J. Pediatr..

[82] Prabhakar S., Zhang X., Goto J., Han S., Lai C., Bronson R., Sena-Esteves M., Ramesh V., Stemmer-Rachamimov A., Kwiatkowski D.J. (2015). Survival benefit and phenotypic improvement by hamartin gene therapy in a tuberous sclerosis mouse brain model. Neurobiol. Dis..

[83] Prabhakar S., Cheah P.S., Zhang X., Zinter M., Gianatasio M., Hudry E., Bronson R.T., Kwiatkowski D.J., Stemmer-Rachamimov A., Maguire C.A. (2019). Long-Term Therapeutic Efficacy of Intravenous AAV-Mediated Hamartin Replacement in Mouse Model of Tuberous Sclerosis Type 1. Mol. Ther. Methods Clin. Dev..

[84] Cheah P.S., Prabhakar S., Yellen D., Beauchamp R.L., Zhang X., Kasamatsu S., Bronson R.T., Thiele E.A., Kwiatkowski D.J., Stemmer-Rachamimov A. (2021). Gene therapy for tuberous sclerosis complex type 2 in a mouse model by delivery of AAV9 encoding a condensed form of tuberin. Sci. Adv..

[85] Mitchell M.J., Billingsley M.M., Haley R.M., Wechsler M.E., Peppas N.A., Langer R. (2021). Engineering precision nanoparticles for drug delivery. Nat. Rev. Drug Discov..

[86] Leandro K., Rufino-Ramos D., Breyne K., Di Ianni E., Lopes S.M., Jorge Nobre R., Kleinstiver B.P., Perdigao P.R.L., Breakefield X.O., Pereira de Almeida L. (2024). Exploring the potential of cell-derived vesicles for transient delivery of gene editing payloads. Adv. Drug Deliv. Rev..

[87] Hu S., Knowlton R.C., Watson B.O., Glanowska K.M., Murphy G.G., Parent J.M., Wang Y. (2018). Somatic Depdc5 deletion recapitulates electroclinical features of human focal cortical dysplasia type IIA. Ann. Neurol..

[88] Klofas L.K., Short B.P., Zhou C., Carson R.P. (2020). Prevention of premature death and seizures in a Depdc5 mouse epilepsy model through inhibition of mTORC1. Hum. Mol. Genet..

[89] Kobayashi T., Minowa O., Sugitani Y., Takai S., Mitani H., Kobayashi E., Noda T., Hino O. (2001). A germ-line Tsc1 mutation causes tumor development and embryonic lethality that are similar, but not identical to, those caused by Tsc2 mutation in mice. Proc. Natl. Acad. Sci. USA.

[90] Meikle L., Talos D.M., Onda H., Pollizzi K., Rotenberg A., Sahin M., Jensen F.E., Kwiatkowski D.J. (2007). A mouse model of tuberous sclerosis: Neuronal loss of Tsc1 causes dysplastic and ectopic neurons, reduced myelination, seizure activity, and limited survival. J. Neurosci..

[91] Carson R.P., Van Nielen D.L., Winzenburger P.A., Ess K.C. (2012). Neuronal and glia abnormalities in Tsc1-deficient forebrain and partial rescue by rapamycin. Neurobiol. Dis..

[92] Bateup H.S., Johnson C.A., Denefrio C.L., Saulnier J.L., Kornacker K., Sabatini B.L. (2013). Excitatory/inhibitory synaptic imbalance leads to hippocampal hyperexcitability in mouse models of tuberous sclerosis. Neuron.

[93] Reith R.M., McKenna J., Wu H., Hashmi S.S., Cho S.H., Dash P.K., Gambello M.J. (2013). Loss of Tsc2 in Purkinje cells is associated with autistic-like behavior in a mouse model of tuberous sclerosis complex. Neurobiol. Dis..

[94] Feliciano D.M., Su T., Lopez J., Platel J.C., Bordey A. (2011). Single-cell Tsc1 knockout during corticogenesis generates tuber-like lesions and reduces seizure threshold in mice. J. Clin. Investig..

[95] Nguyen L.H., Mahadeo T., Bordey A. (2019). mTOR Hyperactivity Levels Influence the Severity of Epilepsy and Associated Neuropathology in an Experimental Model of Tuberous Sclerosis Complex and Focal Cortical Dysplasia. J. Neurosci..

[96] Blair J.D., Hockemeyer D., Bateup H.S. (2018). Genetically engineered human cortical spheroid models of tuberous sclerosis. Nat. Med..

[97] Costa V., Aigner S., Vukcevic M., Sauter E., Behr K., Ebeling M., Dunkley T., Friedlein A., Zoffmann S., Meyer C.A. (2016). mTORC1 Inhibition Corrects Neurodevelopmental and Synaptic Alterations in a Human Stem Cell Model of Tuberous Sclerosis. Cell Rep..

[98] Li Y., Muffat J., Omer A., Bosch I., Lancaster M.A., Sur M., Gehrke L., Knoblich J.A., Jaenisch R. (2017). Induction of Expansion and Folding in Human Cerebral Organoids. Cell Stem Cell.

[99] Iffland P.H., Barnes A.E., Baybis M., Crino P.B. (2020). Dynamic analysis of 4E-BP1 phosphorylation in neurons with Tsc2 or Depdc5 knockout. Exp. Neurol..

[100] Jinek M., Chylinski K., Fonfara I., Hauer M., Doudna J.A., Charpentier E. (2012). A programmable dual-RNA-guided DNA endonuclease in adaptive bacterial immunity. Science.

[101] Ran F.A., Hsu P.D., Wright J., Agarwala V., Scott D.A., Zhang F. (2013). Genome engineering using the CRISPR-Cas9 system. Nat. Protoc..

[102] Wyman C., Kanaar R. (2006). DNA double-strand break repair: All’s well that ends well. Annu. Rev. Genet..

[103] Murugan A.K. (2019). mTOR: Role in cancer, metastasis and drug resistance. Semin. Cancer Biol..

[104] Madsen R.R., Vanhaesebroeck B. (2020). Cracking the context-specific PI3K signaling code. Sci. Signal..

[105] Redenbaugh V., Coulter T. (2021). Disorders Related to PI3Kdelta Hyperactivation: Characterizing the Clinical and Immunological Features of Activated PI3-Kinase Delta Syndromes. Front. Pediatr..

[106] Shao W., Azam Z., Guo J., To S.S.T. (2022). Oncogenic potential of PIK3CD in glioblastoma is exerted through cytoskeletal proteins PAK3 and PLEK2. Lab. Investig..

[107] Zhong Y., Zhou X., Guan K.L., Zhang J. (2022). Rheb regulates nuclear mTORC1 activity independent of farnesylation. Cell Chem. Biol..

[108] Manning B.D., Toker A. (2017). AKT/PKB Signaling: Navigating the Network. Cell.

[109] Komor A.C., Kim Y.B., Packer M.S., Zuris J.A., Liu D.R. (2016). Programmable editing of a target base in genomic DNA without double-stranded DNA cleavage. Nature.

[110] Chen P.J., Liu D.R. (2023). Prime editing for precise and highly versatile genome manipulation. Nat. Rev. Genet..

[111] Nelson J.W., Randolph P.B., Shen S.P., Everette K.A., Chen P.J., Anzalone A.V., An M., Newby G.A., Chen J.C., Hsu A. (2022). Engineered pegRNAs improve prime editing efficiency. Nat. Biotechnol..

[112] Rees H.A., Liu D.R. (2018). Base editing: Precision chemistry on the genome and transcriptome of living cells. Nat. Rev. Genet..

[113] Fan Q., Aksoy O., Wong R.A., Ilkhanizadeh S., Novotny C.J., Gustafson W.C., Truong A.Y., Cayanan G., Simonds E.F., Haas-Kogan D. (2017). A Kinase Inhibitor Targeted to mTORC1 Drives Regression in Glioblastoma. Cancer Cell.

[114] Xie J., Wang X., Proud C.G. (2016). mTOR inhibitors in cancer therapy. F1000Research.

[115] Yang G., Francis D., Krycer J.R., Larance M., Zhang Z., Novotny C.J., Diaz-Vegas A., Shokat K.M., James D.E. (2021). Dissecting the biology of mTORC1 beyond rapamycin. Sci. Signal.

[116] Almacellas Barbanoj A., Graham R.T., Maffei B., Carpenter J.C., Leite M., Hoke J., Hardjo F., Scott-Solache J., Chimonides C., Schorge S. (2024). Anti-seizure gene therapy for focal cortical dysplasia. Brain.

[117] Lee S.H.S., Lee J.Y., Choi J.S., Kim H.J., Kim J., Cha S., Lee K.J., Woo H.N., Park K., Lee H. (2022). mTOR inhibition as a novel gene therapeutic strategy for diabetic retinopathy. PLoS ONE.

[118] Cha S., Seo W.I., Woo H.N., Kim H.J., Lee S.H.S., Kim J., Choi J.S., Park K., Lee J.Y., Lee B.J. (2022). AAV expressing an mTOR-inhibiting siRNA exhibits therapeutic potential in retinal vascular disorders by preserving endothelial integrity. FEBS Open Bio.

[119] Karar J., Maity A. (2011). PI3K/AKT/mTOR Pathway in Angiogenesis. Front. Mol. Neurosci..

[120] Wang D., Tai P.W.L., Gao G. (2019). Adeno-associated virus vector as a platform for gene therapy delivery. Nat. Rev. Drug Discov..

[121] Goncalves M.A. (2005). Adeno-associated virus: From defective virus to effective vector. Virol. J..

[122] Tanenhaus A., Stowe T., Young A., McLaughlin J., Aeran R., Lin I.W., Li J., Hosur R., Chen M., Leedy J. (2022). Cell-Selective Adeno-Associated Virus-Mediated SCN1A Gene Regulation Therapy Rescues Mortality and Seizure Phenotypes in a Dravet Syndrome Mouse Model and Is Well Tolerated in Nonhuman Primates. Hum. Gene Ther..

[123] Sweeney N.P., Vink C.A. (2021). The impact of lentiviral vector genome size and producer cell genomic to gag-pol mRNA ratios on packaging efficiency and titre. Mol. Ther. Methods Clin. Dev..

[124] Parr-Brownlie L.C., Bosch-Bouju C., Schoderboeck L., Sizemore R.J., Abraham W.C., Hughes S.M. (2015). Lentiviral vectors as tools to understand central nervous system biology in mammalian model organisms. Front. Mol. Neurosci..

[125] Kotin R.M. (2011). Large-scale recombinant adeno-associated virus production. Hum. Mol. Genet..

[126] Melin E., Andersson M., Gotzsche C.R., Wickham J., Huang Y., Szczygiel J.A., Boender A., Christiansen S.H., Pinborg L., Woldbye D.P.D. (2023). Combinatorial gene therapy for epilepsy: Gene sequence positioning and AAV serotype influence expression and inhibitory effect on seizures. Gene Ther..

[127] Di Berardino C., Massimino L., Ungaro F., Colasante G. (2024). Gene therapy for Dravet syndrome: Promises and impact on disease trigger and secondary modifications. Rare Dis. Orphan Drugs J..

[128] Ding J., Li X., Tian H., Wang L., Guo B., Wang Y., Li W., Wang F., Sun T. (2021). SCN1A Mutation-Beyond Dravet Syndrome: A Systematic Review and Narrative Synthesis. Front. Neurol..

[129] Yu Z., Zhang M., Luo B., Jing H., Yu Y., Wang S., Luo S. (2020). Lrp4 in hippocampal astrocytes serves as a negative feedback factor in seizures. Cell Biosci..

[130] Ng J., Barral S., De La Fuente Barrigon C., Lignani G., Erdem F.A., Wallings R., Privolizzi R., Rossignoli G., Alrashidi H., Heasman S. (2021). Gene therapy restores dopamine transporter expression and ameliorates pathology in iPSC and mouse models of infantile parkinsonism. Sci. Transl. Med..

[131] Huang S. (2020). mTOR Signaling in Metabolism and Cancer. Cells.

[132] Pakola S.A., Peltola K.J., Clubb J.H.A., Jirovec E., Haybout L., Kudling T.V., Alanko T., Korpisaari R., Juteau S., Jaakkola M. (2024). Safety, Efficacy, and Biological Data of T-Cell-Enabling Oncolytic Adenovirus TILT-123 in Advanced Solid Cancers from the TUNIMO Monotherapy Phase I Trial. Clin. Cancer Res..

[133] He Y., Huang X., Li X., Liu H., Liu M., Tao J., Shan Y., Raza H.K., Liu Y., Zhong W. (2024). Preliminary efficacy and safety of YSCH-01 in patients with advanced solid tumors: An investigator-initiated trial. J. Immunother. Cancer.

[134] Haas A.R., Tanyi J.L., O’Hara M.H., Gladney W.L., Lacey S.F., Torigian D.A., Soulen M.C., Tian L., McGarvey M., Nelson A.M. (2019). Phase I Study of Lentiviral-Transduced Chimeric Antigen Receptor-Modified T Cells Recognizing Mesothelin in Advanced Solid Cancers. Mol. Ther..

[135] Leone P., Janson C.G., Bilianuk L., Wang Z., Sorgi F., Huang L., Matalon R., Kaul R., Zeng Z., Freese A. (2000). Aspartoacylase gene transfer to the mammalian central nervous system with therapeutic implications for Canavan disease. Ann. Neurol..

[136] Crystal R.G., Sondhi D., Hackett N.R., Kaminsky S.M., Worgall S., Stieg P., Souweidane M., Hosain S., Heier L., Ballon D. (2004). Clinical protocol. Administration of a replication-deficient adeno-associated virus gene transfer vector expressing the human CLN2 cDNA to the brain of children with late infantile neuronal ceroid lipofuscinosis. Hum. Gene Ther..

[137] McPhee S.W., Janson C.G., Li C., Samulski R.J., Camp A.S., Francis J., Shera D., Lioutermann L., Feely M., Freese A. (2006). Immune responses to AAV in a phase I study for Canavan disease. J. Gene Med..

[138] Worgall S., Sondhi D., Hackett N.R., Kosofsky B., Kekatpure M.V., Neyzi N., Dyke J.P., Ballon D., Heier L., Greenwald B.M. (2008). Treatment of late infantile neuronal ceroid lipofuscinosis by CNS administration of a serotype 2 adeno-associated virus expressing CLN2 cDNA. Hum. Gene Ther..

[139] Tong J., Buch S., Yao H., Wu C., Tong H.I., Wang Y., Lu Y. (2014). Monocytes-derived macrophages mediated stable expression of human brain-derived neurotrophic factor, a novel therapeutic strategy for neuroAIDS. PLoS ONE.

[140] Sessa M., Lorioli L., Fumagalli F., Acquati S., Redaelli D., Baldoli C., Canale S., Lopez I.D., Morena F., Calabria A. (2016). Lentiviral haemopoietic stem-cell gene therapy in early-onset metachromatic leukodystrophy: An ad-hoc analysis of a non-randomised, open-label, phase 1/2 trial. Lancet.

[141] Huang L., Wan J., Wu Y., Tian Y., Yao Y., Yao S., Ji X., Wang S., Su Z., Xu H. (2021). Challenges in adeno-associated virus-based treatment of central nervous system diseases through systemic injection. Life Sci..

[142] Puhl D.L., D’Amato A.R., Gilbert R.J. (2019). Challenges of gene delivery to the central nervous system and the growing use of biomaterial vectors. Brain Res. Bull..

[143] Lentz T.B., Gray S.J., Samulski R.J. (2012). Viral vectors for gene delivery to the central nervous system. Neurobiol. Dis..

[144] Spunde K., Korotkaja K., Zajakina A. (2022). Recombinant Viral Vectors for Therapeutic Programming of Tumour Microenvironment: Advantages and Limitations. Biomedicines.

[145] Xie L., Han Y., Liu Y., Zhou Y., Yu J., von Brunn A., Lei J. (2023). Viral vector-based cancer treatment and current clinical applications. MedComm Oncol..

[146] Lu X., Zhang M., Li G., Zhang S., Zhang J., Fu X., Sun F. (2023). Applications and Research Advances in the Delivery of CRISPR/Cas9 Systems for the Treatment of Inherited Diseases. Int. J. Mol. Sci..

[147] Tsuchida C.A., Wasko K.M., Hamilton J.R., Doudna J.A. (2024). Targeted nonviral delivery of genome editors in vivo. Proc. Natl. Acad. Sci. USA.

[148] Aljabali A.A.A., El-Tanani M., Tambuwala M.M. (2024). Principles of CRISPR-Cas9 technology: Advancements in genome editing and emerging trends in drug delivery. J. Drug Deliv. Sci. Technol..

[149] Dubey S., Chen Z., Jiang Y.J., Talis A., Molotkov A., Ali A., Mintz A., Momen-Heravi F. (2024). Small extracellular vesicles (sEVs)-based gene delivery platform for cell-specific CRISPR/Cas9 genome editing. Theranostics.

[150] Malik S., Muhammad K., Waheed Y. (2023). Emerging Applications of Nanotechnology in Healthcare and Medicine. Molecules.

[151] Malik S., Muhammad K., Waheed Y. (2023). Nanotechnology: A Revolution in Modern Industry. Molecules.

[152] Villa F., Quarto R., Tasso R. (2019). Extracellular Vesicles as Natural, Safe and Efficient Drug Delivery Systems. Pharmaceutics.

[153] Sun M., Zhang H., Liu J., Chen J., Cui Y., Wang S., Zhang X., Yang Z. (2024). Extracellular Vesicles: A New Star for Gene Drug Delivery. Int. J. Nanomed..

[154] Kumar M.A., Baba S.K., Sadida H.Q., Marzooqi S.A., Jerobin J., Altemani F.H., Algehainy N., Alanazi M.A., Abou-Samra A.B., Kumar R. (2024). Extracellular vesicles as tools and targets in therapy for diseases. Signal Transduct. Target Ther..

[155] Zanirati G., Dos Santos P.G., Alcara A.M., Bruzzo F., Ghilardi I.M., Wietholter V., Xavier F.A.C., Goncalves J.I.B., Marinowic D., Shetty A.K. (2024). Extracellular Vesicles: The Next Generation of Biomarkers and Treatment for Central Nervous System Diseases. Int. J. Mol. Sci..

[156] Chenthamara D., Subramaniam S., Ramakrishnan S.G., Krishnaswamy S., Essa M.M., Lin F.H., Qoronfleh M.W. (2019). Therapeutic efficacy of nanoparticles and routes of administration. Biomater. Res..

[157] Yusuf A., Almotairy A.R.Z., Henidi H., Alshehri O.Y., Aldughaim M.S. (2023). Nanoparticles as Drug Delivery Systems: A Review of the Implication of Nanoparticles’ Physicochemical Properties on Responses in Biological Systems. Polymers.

[158] Wang H., Qin L., Zhang X., Guan J., Mao S. (2022). Mechanisms and challenges of nanocarriers as non-viral vectors of therapeutic genes for enhanced pulmonary delivery. J. Control. Release.

[159] Witika B.A., Makoni P.A., Matafwali S.K., Chabalenge B., Mwila C., Kalungia A.C., Nkanga C.I., Bapolisi A.M., Walker R.B. (2020). Biocompatibility of Biomaterials for Nanoencapsulation: Current Approaches. Nanomaterials.

[160] Kunzmann A., Andersson B., Thurnherr T., Krug H., Scheynius A., Fadeel B. (2011). Toxicology of engineered nanomaterials: Focus on biocompatibility, biodistribution and biodegradation. Biochim. Biophys. Acta.

[161] Jia Y., Jiang Y., He Y., Zhang W., Zou J., Magar K.T., Boucetta H., Teng C., He W. (2023). Approved Nanomedicine against Diseases. Pharmaceutics.

[162] Akombaetwa N., Ilangala A.B., Thom L., Memvanga P.B., Witika B.A., Buya A.B. (2023). Current Advances in Lipid Nanosystems Intended for Topical and Transdermal Drug Delivery Applications. Pharmaceutics.

[163] Patra J.K., Das G., Fraceto L.F., Campos E.V.R., Rodriguez-Torres M.D.P., Acosta-Torres L.S., Diaz-Torres L.A., Grillo R., Swamy M.K., Sharma S. (2018). Nano based drug delivery systems: Recent developments and future prospects. J. Nanobiotechnol..

[164] Yao Y., Zhou Y., Liu L., Xu Y., Chen Q., Wang Y., Wu S., Deng Y., Zhang J., Shao A. (2020). Nanoparticle-Based Drug Delivery in Cancer Therapy and Its Role in Overcoming Drug Resistance. Front. Mol. Biosci..

[165] Huang X., He T., Liang X., Xiang Z., Liu C., Zhou S., Luo R., Bai L., Kou X., Li X. (2024). Advances and applications of nanoparticles in cancer therapy. MedComm Oncol..

[166] Wang B., Hu S., Teng Y., Chen J., Wang H., Xu Y., Wang K., Xu J., Cheng Y., Gao X. (2024). Current advance of nanotechnology in diagnosis and treatment for malignant tumors. Signal Transduct. Target Ther..

[167] Gavas S., Quazi S., Karpinski T.M. (2021). Nanoparticles for Cancer Therapy: Current Progress and Challenges. Nanoscale Res. Lett..

[168] Fathizadeh H., Afshar S., Masoudi M.R., Gholizadeh P., Asgharzadeh M., Ganbarov K., Kose S., Yousefi M., Kafil H.S. (2021). SARS-CoV-2 (Covid-19) vaccines structure, mechanisms and effectiveness: A review. Int. J. Biol. Macromol..

[169] Polack F.P., Thomas S.J., Kitchin N., Absalon J., Gurtman A., Lockhart S., Perez J.L., Perez Marc G., Moreira E.D., Zerbini C. (2020). Safety and Efficacy of the BNT162b2 mRNA Covid-19 Vaccine. N. Engl. J. Med..

[170] Bonvalot S., Rutkowski P.L., Thariat J., Carrere S., Ducassou A., Sunyach M.P., Agoston P., Hong A., Mervoyer A., Rastrelli M. (2019). NBTXR3, a first-in-class radioenhancer hafnium oxide nanoparticle, plus radiotherapy versus radiotherapy alone in patients with locally advanced soft-tissue sarcoma (Act.In.Sarc): A multicentre, phase 2-3, randomised, controlled trial. Lancet Oncol.

[171] Urits I., Swanson D., Swett M.C., Patel A., Berardino K., Amgalan A., Berger A.A., Kassem H., Kaye A.D., Viswanath O. (2021). Correction to: A Review of Patisiran (ONPATTRO(R)) for the Treatment of Polyneuropathy in People with Hereditary Transthyretin Amyloidosis. Neurol. Ther..

[172] Preda A., Shapiro B.B. (2020). A safety evaluation of aripiprazole in the treatment of schizophrenia. Expert Opin. Drug Saf..

[173] Anselmo A.C., Mitragotri S. (2019). Nanoparticles in the clinic: An update. Bioeng. Transl. Med..

[174] Drummond D.C., Noble C.O., Guo Z., Hong K., Park J.W., Kirpotin D.B. (2006). Development of a highly active nanoliposomal irinotecan using a novel intraliposomal stabilization strategy. Cancer Res..

[175] Vergote I., Bergfeldt K., Franquet A., Lisyanskaya A.S., Bjermo H., Heldring N., Buyse M., Brize A. (2020). A randomized phase III trial in patients with recurrent platinum sensitive ovarian cancer comparing efficacy and safety of paclitaxel micellar and Cremophor EL-paclitaxel. Gynecol. Oncol..

[176] Zong Y., Wu J., Shen K. (2017). Nanoparticle albumin-bound paclitaxel as neoadjuvant chemotherapy of breast cancer: A systematic review and meta-analysis. Oncotarget.

[177] Kaye A.D., Armstead-Williams C., Hyatali F., Cox K.S., Kaye R.J., Eng L.K., Farooq Anwar M.A., Patel P.V., Patil S., Cornett E.M. (2020). Exparel for Postoperative Pain Management: A Comprehensive Review. Curr. Pain Headache Rep..

[178] Du Y., Liu Y., Hu J., Peng X., Liu Z. (2023). CRISPR/Cas9 systems: Delivery technologies and biomedical applications. Asian J. Pharm. Sci..

[179] Li T., Yang Y., Qi H., Cui W., Zhang L., Fu X., He X., Liu M., Li P.F., Yu T. (2023). CRISPR/Cas9 therapeutics: Progress and prospects. Signal Transduct. Target Ther..

[180] Razi Soofiyani S., Baradaran B., Lotfipour F., Kazemi T., Mohammadnejad L. (2013). Gene therapy, early promises, subsequent problems, and recent breakthroughs. Adv. Pharm. Bull..

[181] Wang C., Pan C., Yong H., Wang F., Bo T., Zhao Y., Ma B., He W., Li M. (2023). Emerging non-viral vectors for gene delivery. J. Nanobiotechnol..

[182] Wallen M., Aqil F., Spencer W., Gupta R.C. (2023). Exosomes as an Emerging Plasmid Delivery Vehicle for Gene Therapy. Pharmaceutics.

[183] Butt M.H., Zaman M., Ahmad A., Khan R., Mallhi T.H., Hasan M.M., Khan Y.H., Hafeez S., Massoud E.E.S., Rahman M.H. (2022). Appraisal for the Potential of Viral and Nonviral Vectors in Gene Therapy: A Review. Genes.

[184] Shintaro F., Shigeru K., Mitsuru H., Koyo N., Ming W., David G. (2013). Chapter 1: Targeted Gene Delivery: Importance of Administration Routes. Novel Gene Therapy Approaches.

[185] Gessler D.J., Tai P.W.L., Li J., Gao G. (2019). Intravenous Infusion of AAV for Widespread Gene Delivery to the Nervous System. Methods Mol. Biol..

[186] Saint-Pol J., Gosselet F., Duban-Deweer S., Pottiez G., Karamanos Y. (2020). Targeting and Crossing the Blood-Brain Barrier with Extracellular Vesicles. Cells.

[187] Hsu C.-Y., Rheima A.M., Kadhim M.M., Ahmed N.N., Mohammed S.H., Abbas F.H., Abed Z.T., Mahdi Z.M., Abbas Z.S., Hachim S.K. (2023). An overview of nanoparticles in drug delivery: Properties and applications. S. Afr. J. Chem. Eng..

[188] Afzal O., Altamimi A.S.A., Nadeem M.S., Alzarea S.I., Almalki W.H., Tariq A., Mubeen B., Murtaza B.N., Iftikhar S., Riaz N. (2022). Nanoparticles in Drug Delivery: From History to Therapeutic Applications. Nanomaterials.

[189] Li J., Wang Q., Xia G., Adilijiang N., Li Y., Hou Z., Fan Z., Li J. (2023). Recent Advances in Targeted Drug Delivery Strategy for Enhancing Oncotherapy. Pharmaceutics.

[190] Karalis V., Bateup H.S. (2021). Current Approaches and Future Directions for the Treatment of mTORopathies. Dev. Neurosci..

[191] Moloney P.B., Cavalleri G.L., Delanty N. (2021). Epilepsy in the mTORopathies: Opportunities for precision medicine. Brain Commun..

[192] Dentel B., Escamilla C.O., Tsai P.T. (2019). Therapeutic Targeting of mTORC2 in mTORopathies. Neuron.

[193] Condon K.J., Orozco J.M., Adelmann C.H., Spinelli J.B., van der Helm P.W., Roberts J.M., Kunchok T., Sabatini D.M. (2021). Genome-wide CRISPR screens reveal multitiered mechanisms through which mTORC1 senses mitochondrial dysfunction. Proc. Natl. Acad. Sci. USA.

[194] Imanaga H., Semba Y., Sasaki K., Setoguchi K., Maniriho H., Yamauchi T., Terasaki T., Hirabayashi S., Nakao F., Nogami J. (2024). Central role of the mTORC1 pathway in glucocorticoid activity against B-ALL cells. Blood Neoplasia.

[195] Coleman M., Pinares-Garcia P., Stephenson S.E., Lee W.S., Kooshavar D., McLean C.A., Howell K.B., Leventer R.J., Reid C.A., Lockhart P.J. (2024). Ectopic HCN4 Provides a Target Biomarker for the Genetic Spectrum of mTORopathies. Neurol. Genet..

[196] Drago D., Foss-Campbell B., Wonnacott K., Barrett D., Ndu A. (2021). Global regulatory progress in delivering on the promise of gene therapies for unmet medical needs. Mol. Ther. Methods Clin. Dev..

[197] Ahmad A., Munawar N., Khan Z., Qusmani A.T., Khan S.H., Jamil A., Ashraf S., Ghouri M.Z., Aslam S., Mubarik M.S. (2021). An Outlook on Global Regulatory Landscape for Genome-Edited Crops. Int. J. Mol. Sci..

